# Assessment of the Eco-Efficiency of the Circular Economy in the Recovery of Cellulose from the Shredding of Textile Waste

**DOI:** 10.3390/polym14071317

**Published:** 2022-03-24

**Authors:** Geraldo Cardoso de Oliveira Neto, Micheline Maia Teixeira, Gabriel Luis Victorino Souza, Valquiria Demarchi Arns, Henrricco Nieves Pujol Tucci, Marlene Amorim

**Affiliations:** 1 Industrial Engineering Post-Graduation Program, Universidade Nove de Julho (UNINOVE), São Paulo 03155-000, Brazil; geraldo.prod@gmail.com (G.C.d.O.N.); micheline.maiateixeira@gmail.com (M.M.T.); gabrielvictorino@uol.com.br (G.L.V.S.); valquiria.demarchi@cocamar.com.br (V.D.A.); henrricco@gmail.com (H.N.P.T.); 2 GOVCOPP & DEGEIT, University of Aveiro, 3810-193 Aveiro, Portugal

**Keywords:** recovery of cellulose, textile fibers, eco-efficiency, circular economy, textile industry

## Abstract

There is a growing demand for the adoption of cyclical processes in the fashion industry. The trends point to the reuse of cellulose from cotton fibres, obtained from industrial waste, as a substitute to the former linear processes of manufacturing, sale, use, and discarding. This study sets up to explore and assess the economic and environmental gains from the mechanical shredding of cellulose in cotton fabrics in a textile company, identifying the circularity associated with the adoption of such methods. The study resorted to a case study methodology building on interviews and observation. For the environmental estimations, the study employed the material intensity factor tool, and for the economic evaluation the study uses the return on investment. The study also offers an estimation of the circularity of the processes that were implemented. The adoption of the mechanical shredding for cotton cellulose generated economic gains of US$11,798,662.98 and a reduction in the environmental impact that amounts to 31,335,767,040.26 kg including the following different compartments: biotic, abiotic, water, air, and erosion. The findings suggest the existence of opportunities for the circular economy in the textile sector of about 99.69%, dissociated to the use of mechanical recycling, while limited by the consumption of electrical energy and lubricants in the recycling process, leading the way to a circular economy.

## 1. Introduction

The elimination of waste and pollution, the circulation of products and materials, and the regeneration of nature, are the key principles underlying the circular economy perspective, which pursues the goal of rebuilding financial, human, and social capital [1]. According to the principles of a circular economy, the production and consumption activities must be organized to preserve the value of the products, components, and materials throughout all of the value chain and the product life cycle [2]. In the textile sector, the circular business models involve the materials, the facilities, the packaging, the garments, and the programs aimed at retuning textile items instead of discarding such items [3]. As such, all of the value chain is included with initiatives in the several phases of design, production, consumption, and waste management [4].

The transfer of linear business models to the circular economy has been gaining room [5], particularly in the last decade as researchers and policy makers have been discussing strategies to meet sustainability goals [6]. The textile industry, and sector specialists, have also been involved in the development of actions to build knowledge for the circular economy while contributing to raising awareness about sustainability [7]. In this context, the circular economy, as a management approach in the food and textile sectors, offers a real opportunity for the reuse of food waste to produce natural fiber that can be used in the production of new textile products [8]. The research work by Costa et al. [7] also offers an important vision about the valorization of proteins which can be recovered by means of chemical and physical processes from milk byproducts and which can have different applications in the textile industry.

It is noteworthy that demographic growth has influenced the availability of resources globally due to the increase in the consumption of disposable goods, leading to potential excessive levels of consumption and the generation of waste [9]. In the manufacturing context, the textile sector raises concerns about the abundant use of natural resources and of fossil derived materials that raise environmental and social problems due to the manufacturing and discarding of textile clothing [10]. For this reason, organizations are adopting strategies to minimize environmental impacts, such as the use of acetate to transform cellulose waste which is present in cardboard, paper, and cotton (therefore, introducing such cellulose waste into new cellulose textile fiber) [11]. Therefore, the potential for the use of this type of waste as a raw material for conventional processes of fiber spinning holds important contributions for the environment and create opportunities for circular economy [12].

The recycling of textile mixtures has raised the awareness about the development of methods that are safe for the environment. In studies that address fabric made with yarn mixing polyester and recycled cotton, some technical evaluation has been conducted about the mechanic recycling of the fibers. Despite the existing functional differences between virgin and used polyester fibers, recycled polyester fibers can be used in adequate fabric, minimizing the production of virgin fiber and contributing to reducing the environmental impact [13]. The process of enzymatic hydrolysis can also be used as a strategy to recycle the mix of polyester and cotton. It involves two stages. The first stage involves a mechanical pre-treatment by means of grinding with the purpose of obtaining short fibers for the textile structure. Then, an alkaline chemical pre-treatment follows wherein the material is exposed into a sodium hydroxide solution for a given amount of time. Despite the fact such a process had not been implemented at an industrial level, it proves to be a valid opportunity for the recycling of mixed fabrics including polyester and cotton (which is aligned with the goals of the circular economy in the textile sector) [14]. Concerning the mixtures of polyester and wool fiber, some experiments involving enzymatic treatment of the mix have also evidenced promising results for the recovery of synthetic fibers, since the textile waste involving mixed materials have aggravated the economic and environmental impacts in the sector [15].

The environmental impacts of the bio recycling method, which recovers polyester fibers from the mixture of polyester and cotton in fabric waste, have been evaluated using the life cycle assessment method (LCA) for the stages of pre-treatment, melt-spinning, and enzymatic hydrolysis. This allowed for the observation that the pre-treatment process contributes with an impact of 60%, while the melt spinning has smaller impacts for the ecosystems (14%) and for human health (15%), and enzymatic hydrolysis affects the quality of the ecosystem (14%) and human health (12%). The results suggest that the bio recycling of textiles creates opportunities for the reduction of environmental impacts [16].

The evaluation of the environmental impact associated to the use of mechanically recycled cotton fiber instead of using virgin cotton fiber was also conducted using the LCA method. The results observed, in the scenarios that were evaluated, highlighted some key aspects such as the denim fabric using cotton fiber as a raw material (53%) and the energy consumed in the e spinning phase (16%). In terms of what concerns the environmental impact, the use of the 100% recycled cotton fiber has the smaller impact. The results of existing studies might encourage textile companies to use recycled raw materials as well as the use of eco-friendly technologies for energy production [5].

It stands out that very often, textile materials are discarded in landfill sites or incinerating plants [17], leading to serious environmental risks [18]. Meanwhile, to reduce environmental impacts, some companies have been adopting strategies to reuse the textile waste [19]. The implementation of recycling reutilization practices for textile waste following the consumption can reduce pollution and the volume of discards in landfills, reducing both the environmental impacts and the costs associated with the discarding activities [20]. Likewise, the separation of large volumes of textile materials through automated processes can also contribute to minimize the production of virgin textile fibers and to the increase in the levels of reutilization and recycling levels [21].

According to studies addressing the use of cotton fiber recycled through mechanical processes instead of virgin cotton fiber, the economic evaluation performed using relative costs led to different results for each scenario. When using 100% recycled cotton fiber, instead of virgin fibers, the cost variation was only of 1%. The utilization of the combined heat and energy plant led to the observation of a reduction in cost around 35 to 40% for each meter of fabric. However, the increase in the consumption of electricity to produce thread with recycled fiber led to an increase in the energy costs of 8% [5].

As such, several studies have demonstrated the existence of efforts to change the perception about the textile waste which is generated throughout the production chain, addressing several good practices that are environmentally friendly [20]. Such practices are related to the development of materials from waste [5,14,19,22,23,24], the recycling of textiles [12,20,25,26,27], and the development of textile fibers from non-textile waste, among others [7,8,9,28]. Table 1 offers an overview of the literature addressing the textile industry and the approaches related to economic and environmental aspects that hold opportunities for the circular economy.

The research conducted by Fidan et al. [5] and Subramanian et al. [16], describes the conduction of environmental assessments using the LCA method. However, these studies do not distinguish those results across departments. None of the existing studies offers an assessment of the environmental impact using the mass intensity factor (MIF) tool, or present evidence about reductions in the volume of waste that is attributed to the recycling, reuse, or the development of new materials. Concerning the economic evaluation of the results, no studies were identified using an economic evaluation that builds on the estimation of the return on investment (ROI). The existing studies only offer a qualitative description of the economic gains. In the study of Fidan et al. [5] the authors used relative costs to describe the economic gains associated with each of the scenarios addressed in the study.

**Figure 1:**
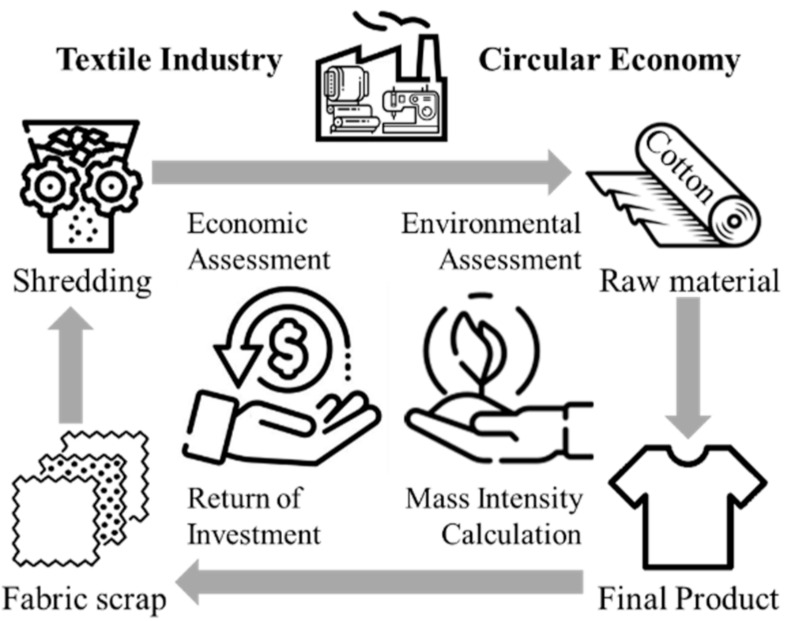
Proposed application of the circular economy in the textile industry.

Therefore, gaps were identified in the literature, which justify the development of this study and to offer a contribution to the following research question: Does the adoption of mechanical splitting of cotton cellulose generate economic and environmental gains and opportunities for the circular economy in the textile industry? This study therefore aims to assess the economic and environmental gains that can be derived from the mechanical splitting of cellulose in cotton fabric in a textile company, and to discuss the opportunities for the circular economy associated with this method (Figure 1).

Among the processes used for recycling of textiles, the mechanical recycling is a process widely adopted. In this process, the grinding or splitting is used to extract the fiber from textile waste either before or after consumption and use [25]. Cotton is one of the most important materials for textile production, but is has raised concerns about its environmental impacts, since its growth and cultivation require large volumes of water and pesticides and its manufacturing involves high levels of energy consumption [5]. Globally the production of fibres has been growing and reaching levels around 100 million of tons per year [17]. A large share of this production is directed to supply the textile industry that has also been registering important levels of production growth [29]. This growth is influenced also by the prevalent fast fashion trends [28]. The fast fashion tendency contributes to the increase in the negative impacts for the environment, which are related to the large volumes of water and energy that are consumed in the production and distribution processes including the extraction of raw materials, the production of textile fibres, the weaving, the dying, washing, and end of life processes [6]. In this context the mechanical splitting of the textile cellulose can offer a promising strategy to obtain environmental and financial gains, contributing to reduce the pressures that the textile sector faces.

## 2. Materials and Methods

### 2.1. Data Collection Methods

**Figure 2:**
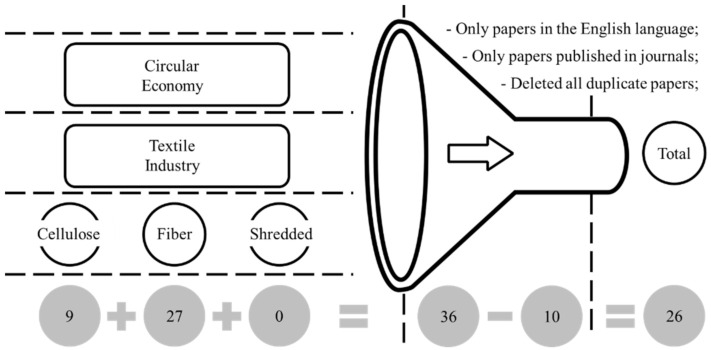
Protocol used for the systematic literature review.

This study addressed the mechanical spliting of cellullose in cotton textiles in the specific context of a textile company, with the purpose of identifying opportunities for circular economy and attaining economic and environmental benefits that are quantifiable. According to Yin [32], the conduction of case studies is recommended when the researcher wants to explore research questions such as “how” and “why”. In addition, according to Eisenhardt [33], the study has characteristics of an exploratory work which investigates a contemporary scenario and addressed a real-world problem. Following the identification of the research gap, the study involved the conduction of a literature review using the following research terms: “circular economy”; “textile industry”; “fiber”; “cellulose”; “shredded” (Figure 2). The databases included in the search where Science Direct, Emerald Insight, Wiley, Taylor & Francis, and Google Scholar. All of the articles were analysed according to the principles proposed by Bryman [34] with the purpose of identifying the theoretical constructs building on content analysis techniques.

**Figure 3:**
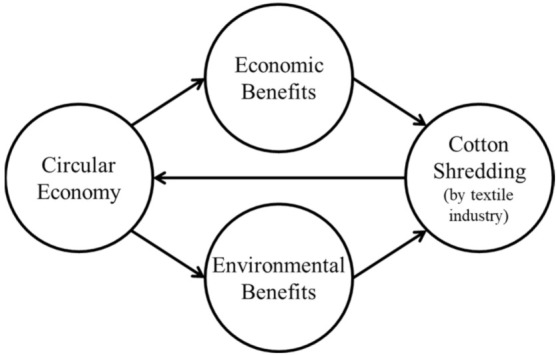
Conceptual model.

The 26 articles identified supported the observed paucity of research in the context of textile industries in the topics related to circular economy, notably for studies addressing the economic and environmental impacts resorting to quantitative and qualitative methods. Building on the existing sources, the following conceptual model was proposed (see Figure 3).

The next phase in the study involved the conduction of field study with the purpose of exploring the theories identifies in the literature towards the managerial practices adopted in the textile industry. To this end, the data collection involved the conduction of semi-structured interviews with the technical managers of the company addressed in the study. According to Bryman [34] and Collins and Hussey [35], this is an adequate approach to collect quantitative data. The process also involved the development of an interview script with a key set of questions to support the conduction of the interviews.

### 2.2. Data Analysis

The data analysis was organized into two steps. First, the environmental aspects were addressed, and next the economic perspective was explored. It is important to highlight that the textile company addressed in the study approached the circular economy by investing in equipment aiming at the reduction of waste. Therefore, it was necessary to consider both the costs and the environmental impacts associated with these changes in the production process following the acquisition of that equipment. To increase the transparency of the impacts being investigated the study adopted a comparative logic approach (i.e., analysing the situation “before” and “after”).

The environmental analysis was performed with the calculation of the mass intensity total (*MIT*), as this is a method that considers biotic compartments (x), abiotic (w), air (z), and water (y) for analysing the environmental impacts of the process focused on in the study [36]. According to Tucci et al. [37], it was necessary to first proceed with the identification of the quantities consumed in the processes and convert these quantities into mass units (*M*). Next, it was necessary to multiply the quantities by the corresponding intensity factor (*IF*) (Equation (1)).

(1) MIF=M×IF

The values for the *IF* are released, and updated regularly, by the Wuppertal Institute. As previously mentioned this study considered the elements of cotton, oil, and electrical energy (Table 2).

Accordingly, it was possible to calculate the mass intensity per compartment (*MIC*) by adding all together (Equation (2)) the three elements considered in the study for each compartment. In other words, it was possible to identify the shares for environmental impacts that were avoided in each compartment.

(2) $$MIC=IF\;\left(residue\;A\;compartment\;w\right)+IF\;\left(residue\;B\;compartment\;w\right)+IF\;\left(residue\;C\;compartment\;w\right)+\ldots$$

Finally, the sim of the *MIC* for each of the compartments let to the total environmental impact that was prevented. This estimation was defined as the mass intensity total (*MIT*) (Equation (3)).

(3) MIT=MICw+MICv+MICz+MICn

It is noteworthy that the study considered the reduction in the use of cotton as raw material by reusing the production waste, as well as the consumption of energy and lubricant oils in the new equipment (which allowed for the reuse of the materials). As such, it was possible to estimate the level of circularity of the company using the equation below.

(4) $$Circularity\;Index=\frac{Mass\;Intensity\;Total\;Before}{Mass\;Intensity\;Total\;After}$$

The economic analysis was performed by calculating the ROI, which has the objective of determining the amount of time that is necessary to cover the debts that were incurred to support the total investments made. By determining the ROI, companies can analyse the economic viability of each innovation, change, or modernization, regardless of the volume of the investment. This is a standard method that is applied across all industries [39,40].

The study considered in the stage “before” the reusing of the textile wastes the direct costs related to the acquisition of raw materials.

In the “after” stage, other costs such as human resources were considered, which grouped costs such as hiring employees, technical training, overtime to adapt the new workflow and other similar costs. In addition, costs with laboratory tests, food, as well as costs with lubricants, electricity, janitorial, maintenance, storage, and insurance, logically affected by the acquisition of new machines, with emphasis on the large increase in insurance costs.

The data collected allowed for the estimation of the depreciation costs for the equipment acquired and the costs related to taxes and social contributions. As such, it was possible to perform a comparative analysis for each year to determine the moment when the accumulated returns would surpass the yearly depreciation and the return period for the investment.

## 3. Results and Discussion

The object of this case study is a cooperative company that started its activities in the 1960s, bringing together 46 agricultural producers who wanted to diversify their products. Cotton was selected as the product that would initiate this diversification. However, the success of the cooperative resulted in the expansion of business. Currently, there are 97 operational units and more than 15,000 members.

In the 1980s, the cooperative opened its yarn industry with the aim of adding value to the cotton delivered by the cooperative members. However, it was in 2011 that the entire industrial park was modernized to increase export volumes, improving the quality of the final product and complying with international standards (mainly from the European market, which are stricter than national standards). Since then, the guide to good practices in the textile sector has been followed, with emphasis on waste reduction programs.

The high levels of demand for virgin fibres carry important impacts for the environment, since their cultivation often involves the use of pesticides. Moreover, along all of the manufacturing process, much waste is generated which are considered by-products with low value.

On the contrary, in the context of the textile industry, we have been observing a growth in the studies concerning the reduction of waste aiming at eliminating or reducing them, according to the principles of the circular economy. One of the avenues for that is the reutilization of cellulose by splitting the remainders of textile cuttings, as well as of new textiles or those that are at the end of their lifecycle, by incorporating them into the production of split thread. Moreover, over the last years new industrial equipment in the textile industry, that have higher capacity, demand fibres with higher intrinsic quality, and this strengthens the challenge for pursuing the goals of circular production.

**Figure 4:**
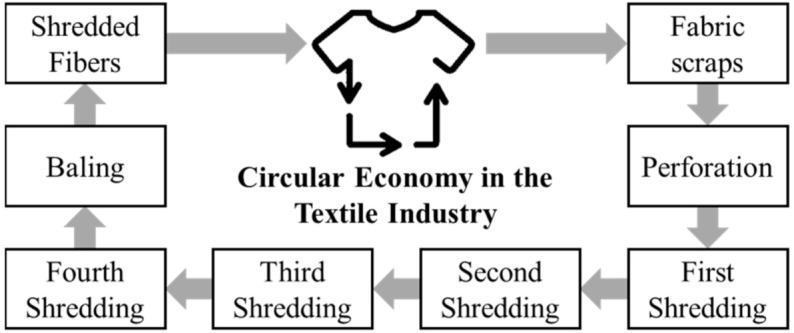
Process for the mechanical recovery of cotton fiber.

In Figure 4 we present the flowchart for the stages involved in the process of mechanic recovery of cellulose by splitting textile material.

The process involves:

✓ The separation of the textile material by color: the separation of the textile scraps is made according to the color and is performed in the textile industry with the purpose of obtaining split fibres with solid colors and with the lowest levels of contamination. The index of contamination involves the mix of scraps with different colors from the main colour.
✓ Perforation: this stage aims to reduce the size of the scraps to a size of approximately 5 cm, to facilitate the shredding process that will follow.
✓ First to fourth shredding: each shredding stage aims to separate the fibres from the scrap. In each step, the separation is improved.
✓ Baling: the shredded fibres are compacted in bale format and are conditioned in bags.

### 3.1. Technical Analysis for the Recovery of Textile Fibres

In the context of the thread industry, which uses shredded fibres as a substitute for virgin cotton fibre, some control points are important such as the measurement of the composition of the mixture bale and the identification of the contamination and the intrinsic quality of the fibres and its thread. Since they originate from the scrap from textiles used to produce clothing, the risks of mixing materials in the moment of separation that is performed in the textile company is very high. This is reflected in the variability of the composition that can be found in the bale for shredded cotton. This creates difficulties for the composition of the textile products, as illustrated in Table 3. In Brazil, the correct identification of the final composition of a textile product is mandatory and subject to fines if carried out incorrectly. The variability that characterizes the composition of shredded textile bale is addressed in the law. The Portaria Inmetro n° 118 of 10 March 2021, states that “UNDEFINED COMPOSITON” or “DIVERSIFIED FIBERS” can only be used for textile products for which the textile composition can be difficult to determine, such as in the case of shredded cotton fibres.

The contamination that exists in the shredded fibre bale, such as parts of buttons, zips, and needles is also an element of difficulty for the production since it reduces the lifetime of the shredding equipment as illustrated in Table 3. IT reduces the efficiency of the shredding and carding as well as the risk of accidents for the workers that handle the materials (including the risk of fire). The reduction in the efficiency of the shredding process is a very important factor to monitor. Also, the reduction in the lifetime of the equipment derived from the metal contamination increases the maintenance costs. Fibres from obtained from shredding processes have an average length and resistance that have a direct impact for the reliability index.

The reliability can be measured according to the formula where:

CI = −322.98 + (2.89 × STR)−(9.02 × MIC) + (43.53 × (UHML)) + (4.29 × UI)

SCI (spinning consistency index)—index of reliability for the fibre. The bigger the value of the index, the better is the quality of the material, and the better the manufacturing performance.

STR (strength)—fibre resistance. This corresponds to the strength measured in grams which is necessary to break a bundle of fibre of 1 tex, measured in gr/tex.

MIC (micronaire)—index for fineness. Measures the linear density (mass/length) through maturity.

UHML (upper half mean length)—average length for the upper half of the 50% of the longer fibres, measured in inches.

UI (uniformity index)—index for the uniformity of the length of the fibre, corresponds to the relationship between the medium length (ML) and the medium length for the upper half of the longer fibres (UHML), measured in %.

The formula for the reliability index is recommended by the manufacturers of equipment for HVI (high volume instrument), that performs the measurement of the information about the intrinsic quality of the cotton feather. There are two main manufacturers globally for this type of equipment: Uster Technologies [41] and Premier Evolvics [42].

A lower level for SCI created difficulties for the processing of the fibres to produce the thread, requiring lower speed from the equipment and resulting in losses in production. Depending on the spinning method that is used to make the thread, there will be a stronger demand for length and resistance of the fibres, notably for threads that are very thin for some fashion segments. One alternative to produce thinner shredded threads is to make a mixture adding some proportion of other fibres such as cotton, polyester, and fibres from recycled materials or from certified or organic materials. Such a mixture helps to increase reliability, easing the process of obtaining threads and reducing losses. In Table 3, a comparative view is offered with examples for the reliability index for a shredded fibre and a virgin fibre.

**Figure 5:**
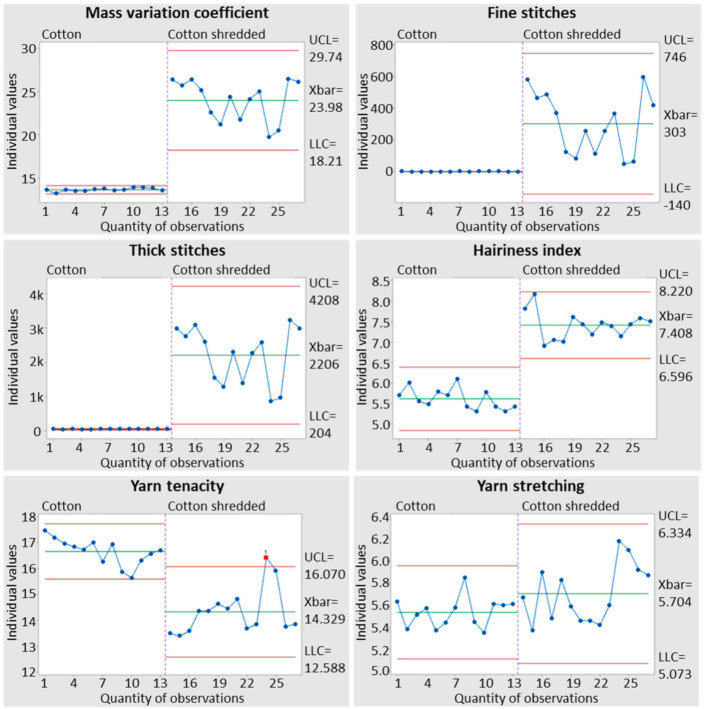
Comparative analysis for thread, produced from 100% cotton and from cotton shred.

Likewise, the thread from shredded fibres has a higher number of irregularities, including the mass coefficient (%), thin spots (−50%), thick spots (+50%), hairiness (%) and tenacity (cN/tex), when compared with virgin fibre threads. The irregularities in threads that are composed by shredded fibres also contribute to difficulties in the production both in shredding and in weaving when producing fabric and knitted fabric. In Figure 5, it is possible to observe a comparison between the thread produced from spinning 100% cotton and shredded cotton.

### 3.2. Assessment of the Environmental Impact from the Implementation of the Process for Recovering Textile Fibres

The company addressed in the study uses 4,600,000 kg of cotton per year to produce fabric, as displayed in Table 4. Before the implementation of the circular economy principles, the company used virgin cotton, carrying a negative environmental impact of: 39,560,000 per kg in the abiotic levels, that stand for global warming, soil, humidity, vegetation etc.; 13,340,000 kg in the abiotic compartment related to life; 31,344,400,000 kg in the air, due to the generated emissions; 12,604,000 kg in water, due to the consumption in the irrigation process; and 23,046,000 kg for erosion due to the cultivation and crops in a linear cycle. Overall, it added up to a total negative environmental impact of 31,432,950,000 kg.

The implementation of the principles of the circular economy led the company to focus on the process of recovering fibres by means of recycling and reuse of scrap from fabric in the textile industry, as well as for fabric that is discarded in the end of its lifetime. This led the company to engage in recycling processes with the goal of recovering 6,223,529.42 kg of fibres. However, 811,764.71 kg of this are non-recoverable and the waste is discarded in an adequate manner. In the process, 5,411,764.71 kg of the fibre is recovered with the original level of quality. The company implemented the process of mechanic recycling for textile waste that consumes 1,148,400 kW/h per year, with the use of perforating equipment, shredding equipment, baling machinery, compacting, and lighting equipment that generate a negative environmental impact of 97,181,901.82 kg when considering all of the compartments (i.e., abiotic, biotic, water, air, and erosion). The company also consumes 192 L of lubricants for the machines, adding to a total negative environmental impact of 1057.92 L.

This study offers a contribution for the advancement of current knowledge about circular economy by describing the evaluation of the environmental impacts for the various compartments (i.e., biotic, abiotic, water, air, and erosion). The values change from a level that was at 31,432,950,000 kg of negative impact before the implementation, and a reduction of the environmental impact amounting to 31,335,767,040.26 after the implementation of the circular economy, in the process of recovery of textile fibres. The existing studies on the topic by Subramanian et al. [16] and Fidan et al. [5] usually use only the mass balance for the process of analysing the lifecycle and do not offer an estimation for the environmental impact. In this context, the contribution of this study can also offer support to the managerial function about the opportunities for the recycling of textile waste by transforming them into fibres for replacing the consumption of cotton.

In the case addressed in the study, a total of 99.69% of circularity was obtained by dividing the 31,335,767,040.26 (corresponding to the reduction in the environmental impact) by 31,432,950,000 kg (corresponding to the negative environmental impact) in relative terms. The data suggest that despite using 100% of recycled fibre in the manufacturing process there was a loss of 97,182,959.74 due to the consumption of energy and lubricants. The valorisation of textile waste is an important point to produce clothing and the recycling alternatives have an important role for the creation of opportunities for circular economy. These findings support the work of Subramanian et al. [16] which states that even if mechanical recycling is the process that is most used for recycling waste from textiles, and which allows for mixing of recycled and virgin fibres, the process also has disadvantages, including the loss of quality in the fibre. As such, mechanical recycling does not generate 100% of circularity due to the productive resources that are consumed in the process (despite the fact that if offers a good contribution for the circular economy). Other technologies exist, such as the process of enzymatic hydrolysis for the treatments of fabric made of polyester and cotton [14] and for mixed fabrics of wool and polyester. These have proved to be an effective textile recycling process, relying on the shredding of fibres, and can bring the fashion industry to a new model of circular economy [15]. The bio-recycling approach also offers technologies that can contribute to the valorisation of the textiles while being environmentally friendly [16].

### 3.3. Economic Evaluation of the Implementation of the Process for Recovering Textile Fibre

Table 5 offers an overview of the cost analysis that allows for the identification of the reduction in the annual cost derived from the circular economy, considering the recycling of textile waste and the recovery of fibre for production. Considering the consumption of cotton for the annual production of 4,600,000 with a cost of US$2.90 per kg, it amounted to US$13,323,272.72. After the implementation of a circular economy, we observed a volume of direct costs of US$264,353.64 that resulted from hiring employees, including four for the operations of recycling and shredding and two for the maintenance of machinery and equipment (plus one manager and one administrative assistant). It is important to highlight the use of bags for conditioning the textile fibres after the recycling process and after being compacted, with the purpose of not using packaging that would generate waste in the textile chain. As such, they were considered an investment and not a direct cost. The indirect costs were also calculated at US$1,031,564.73, considering the costs of lubricants “synthetic emulsifiable oil” for the equipment, the consumption of electric energy, lab tests, food, storage costs, insurance, and indirect costs with the maintenance of machinery, equipment, and facilities. Also, and according to what was mentioned in the environmental assessment, the need to discard 811,764.71 kg of fibre added up to a loss of US$228,691.48 in the recycling due to the impurities that contaminate the cotton fibres. As such, the total cost amounted to US$1,524,609.75, considering the total of direct costs, the indirect costs, and the losses associated with the discarding of waste.

Replacing the purchase of virgin cotton with the utilization of recycled textile fibres led to a cost reduction of US$ 11,798,662.98, demonstrating a relevant pathway for textile companies to adopt the circular economy through the recycling and recovery of textile fibres. In this way, the company no longer purchases virgin cotton and contributes to the sustainability of the ecosystem while simultaneously reducing costs.

The company had to invest US$463,646 in infrastructures, including the acquisition of perforating equipment, shredding equipment, compacting machines, and 948 bags with a capacity of 1 m³/1000 kg to protect the textile fibres from contamination and fungus. The reduction in annual costs amounted to US$11,798,662.98, as detailed in Table 5. As such leaving out the taxes and the values for depreciation, it is possible to observe a reduction in net cost of US$8,931,747 and a return on the capital invested of 30 days, suggesting a profitable opportunity for the textile industry, as displayed in Table 6. The study contributes to the advancement of theory by being the first work that evaluated costs and estimates the return on investment exploring real data from organizational experiences.

No other studies were identified offering an economic evaluation and using the estimation of the returns on investment. The existing studies are limited to the description of the economic gains in a descriptive and qualitative manner. Previously, only the work of Fidan et al. [5] used relative costs to offer an approximate description of the economic gains for the scenarios explored in the study.

## 4. Conclusions

The textile industry has an important share in the emission of CO_2_ and is a big contributor for the environmental pollution. The textile and clothing sector generate tons of waste in the form of scrap that result from cutting of fabric, and most of the time these have as their final destination disposal in landfills. Moreover, in recent years the demand triggered by fast fashion trends increased the speed of production to meet the release of new collections and has been contributing to an excess in the volume of sold products, as well as unsold items that contribute to the discarding of textile materials in harmful ways for the environment. The textile chain carries social and environmental impacts. Despite contributing to the creation of jobs and income, it also contributes to environmental pollution, significant use of water resources, and the exploitation of human resources. Against this background, we are witnessing changes in consumer behaviour, as consumers are demanding products that are more sustainable and socially correct. There is a growing trend that has been acknowledged by fashion influencers for the demand of cyclical processes that make the reuse of cellulose from cotton fibres that are obtained from industrial waste in order to substitute the former linear processes of manufacturing, sale, use, and discarding.

According to the sustainable development goals set in the Agenda 2030 by the UN, the five guiding principles (people, planet, prosperity, peace, and partnership) will promote sustainability and innovation through the adoption of technologies that contribute for the implementation of industrial processes that are environmentally adequate. Accordingly, this study addresses the case of reuse of cellulose obtained from the waste generated in the textile manufacturing processes by producing shredded thread. The study supports the notion that the adoption of mechanical shredding for cotton cellulose allows for achieving economic gains of US$11,798,662.98 and a reduction in the environmental impacts of 31,335,767,040.26 kg when adding the several compartments (biotic, abiotic, water air, and erosion). These findings suggest opportunities for the circular economy in the textile sector around 99.69%, from the use of mechanical recycling, the consumption of energy, and lubricant in the recycling process, as an example of a way forward for the circular economy. Future research work should explore other recycling processes for textile fibres and explore both their impacts and circularity.

## Figures and Tables

**Table 1:** Overview of existing literature focusing on the textile industry and the approaches for economic and environmental gains, and opportunities for the circular economy.

			Environmental Approach		Economic Approach	
References	Year	General Approach and Opportunities for the Circular Economy	Qualitative	Quantitative	Qualitative	Quantitative
[27]	2016	Production of synthetic geotextiles non weaved with recycled textile waste	X			
[12]	2019	Utilization of chemical processes for separating cotton and polyester from textile waste	X			
[29]	2019	Methods for textile recycling	X		X	
[21]	2020	Automatic separation of large volumes of textile waste	X		X	
[13]	2020	Evaluation of the properties of recycled thread made of polyester and cotton	X			
[16]	2020	Evaluation of the lifecycle of textile bio recycling	X	X		
[15]	2020	Enzymatic processes for the selective digestion of wool fibres and mixed wool and polyester fibre	X			
[17]	2020	Project TEX2MAT for the recycling of materials from textile flows and selected multi-materials	X			
[10]	2021	Enzymatic and biological processes	X			
[14]	2021	Removing cotton from textile mixture of cotton and PET through enzymatic hydrolysis	X			
[9]	2021	Creation of protein fibres regenerated from waste in the food industry	X			
[18]	2021	Incorporation of dyed cotton flakes in the polypropylene	X		X	
[22]	2021	Utilization of hemp as a natural fibre for the development of green label products	X			
[23]	2021	Replacing polyester by three biological alternatives	X			
[5]	2021	Investigating the contribution of using fibre of mechanical recycled cotton instead of virgin fibre	X	X	X	X
[20]	2021	Valorisation of textile waste for end-of-life products	X		X	
[30]	2021	Investigating the consumption of electric energy to produce cotton clothes	X			
[28]	2021	Investigation of the technological innovation in fibres produced from proteins	X			
[25]	2021	Identification and valorisation of solid waste from textiles, pre-treatment methods	X			
[26]	2021	Recovery of waste in the Nazi period with direct influence in industry sectors, including textile	X			
[31]	2021	Use of iron nitrate to modify the leftovers of cotton in nature and to absorb the back reactive colorant	X			
[7]	2021	Recovery of milk proteins and by-products by chemical and physical processes for applications in non-food industries	X			
[19]	2021	Scientific research addressing the application of recycled fibres	X			
[8]	2021	Utilization of food industry waste to produce bio-textiles	X			
[24]	2021	Development of t a methodology for the fabrication of textile composites with cellulose regenerated from textile waste	X			
[6]	2022	Influences of digital solutions in the textile industry with opportunities for the circular economy	X			

**Table 2:** Impact factors.

*IF*	Abiotic	Biotic	Water	Air	Erosion
Cotton Fiber	8.6	2.9	6.814	2.74	5.01
Energy	2.67	−	37.92	0.64	−
Synthetic Oil	1.22	−	4.28	0.01	−

Source: Wuppertal Institute [38].

**Table 3:** Reliability index.

Material	UHML (in)	Micronaire	STR (gf/Tex)	UI (%)	Spinning Consistency Index (SCI)
Cotton lot	1.2	4.32	31.55	81.14	130
Shredded cotton lot	0.83	4.47	27.84	58.74	5

**Table 4:** Environmental assessment of the process for recovering textile fibers.

Components	Annual Consumption (kg/kWh)	Compartiments/Unit (kg/kWh)					MIT-Mass Intensity Total
		Abiotic	Biotic	Air	Water	Erosion	
“Before” the Adoption of Circular Economy							
Cotton fiber		8.6	2.9	6.814	2.74	5.01	31,432,950,000.00
	4,600,000	39,560,000.00	13,340,000.00	31,344,400,000.00	12,604,000.00	23,046,000.00	
MIC-Mass intensity per compartment-Cotton fiber		39,560,000.00	13,340,000.00	31,344,400,000.00	12,604,000.00	23,046,000.00	31,432,950,000.00
“After” the Implementation of Circular Economy							
Fabric Fiber Recovery							
Reduction from the reuse (cotton shredding)		8.6	2.9	6.814	2.74	5.01	36,979,941,204.61
	5,411,764.71	46,541,176.51	15,694,117.66	36,875,764,733.94	14,828,235.31	27,112,941.20	
Waste discarding (cotton shredding)		8.6	2.9	6.814	2.74	5.01	5,546,991,204.61
	811,764.71	6,981,176.51	2,354,117.66	5,531,364,733.94	2,224,235.31	4,066,941.20	
MIC-Mass intensity per compartment-fiber recovery		39,560,000.00	13,340,000.00	31,344,400,000.00	12,604,000.00	23,046,000.00	31,432,950,000.00
Energy Consumption							
Energy(perforating equipment)		2.67		37.92	0.64		10,802,234.63
	156.975	419,123.25		5,952,492.00	100,464.00		
Energy(shredding equipment)		2.67		37.92	0.64		25,925,363.10
	376.740	1,005,895.80		14,285,980.80	241,113.60		
Energy(shredding equipment)		2.67		37.92	0.64		7,719,997.01
	112.185	299,533.42		4,254,047.62	71,798.27		
Energy(Compacting)		2.67		37.92	0.64		43,945,255.90
	418.600	1,117,662.00		15,873,312.00	267,904.00		
Energy (Lighting)	83.720	2.67		37.92	0.64		8,789,051.18
		223,532.40		3,174,662.40	53,580.80		
MIC-Mass Intensity Per Compartment-Energy Consumption		3,065,746.87		43,540,494.82	734,860.67		97,181,901.82
Consuption of Lubricant for the Equipment							
Synthetic emulsifiable oil	192	1.22		4.28	0.01		1057.92
		234.24		821.76	1.92		
MIC-Mass Intensity per Compartment-Lubricants Consumption		234.24		821.76	1.92		1057.92
MIC-Mass Intensity per Compartment-Energy And Lubricants Consumption		3,065,981.11		43,541,316.58	734,862.59		97,182,959.74
MIT-Mass Intensity Total		36,494,018.89	13,340,000.00	31,300,858,683.42	11,869,137.41	23,046,000.00	31,335,767,040.26
Circularity Index			Before	31,432,950,000.00	After	31,335,767,040.00	99.69%

**Table 5:** Assessment of costs from the process of recovering textile fibres.

Costs (US$) before the Adoption of the Circular Economy	
Utilization of virgin cotton for yearly production	4,600,000
Average price for virgin cotton, per kg	2.90
Costs for the purchase of virgin cotton	13,323,272.72
Costs (US$) After the adoption of the Circular Economy	
Direct Costs (Year)	264,353.64
Fixed Human Resources	264,353.64
Indirect Costs (Year)	1,031,564.73
Lubricants	4809.09
Electric Energy	196,664.64
Lab tests	13,590.91
Food	117,090.91
Storage and Insurance	417,136.36
Indirect Costs for the recycling process	749,291.91
Maintenance of Machinery and Equipment	101,409.09
Professional Services for Machines and Equipment	87,818.18
Facilities Maintenance	93,045.45
Indirect Maintenance Costs	282,272.73
Losses from recycling, leading to waste	228,691.48
Total Costs (US$)	1,524,609.75
Reduction in annual costs (US$)	11,798,662.98

**Table 6:** Assessment of return on investment.

Investiment in equipment	463,646					
Depreciation period (years)	10					
Annual Depreciation	46,365					
Annual Cost Reduction	11,798,663					
Annual Depreciation	−46,365					
Basis for Determining Income Tax (IR)	11,752,298					
IRPJ + CSLL (Social Contribution)	24.0%					
Value for IR + CSSL (Annual)	−2,820,552					
Net Cost Reduction (Annual)	8,931,747					
Net Cost Reduction (Annual)	8,931,747					
Depreciation (Annual)	46,365					
Income (Annual)	8,978,111					
Cash Flow	Year 0	Year 1	Year 2	Year 3	Year 4	Year 5
Investiment	−463,646					
Income Flow (Annual)		8,978,111	8,978,111	8,978,111	8,978,111	8,978,111
Total Cash Flow	−463,646	8,978,111	8,978,111	8,978,111	8,978,111	8,978,111
ROI or TIR	1936.4%	per year				
Payback Discounted at 15% per year	0.08	years				
